# Genome-Wide Identification and Characterization of the *Rboh* Gene Family in *Populus trichocarpa* Reveals *PtrRbohB* Positively Regulates Adventitious Root Development

**DOI:** 10.3390/ijms27208982

**Published:** 2026-10-10

**Authors:** Run Shi, Yanping Ding, Peng Guo, Fude Shang, Shenquan Cao

**Affiliations:** College of Life Sciences, Henan Agricultural University, Zhengzhou 450046, China; srshirun@163.com (R.S.);

**Keywords:** woody plants, adventitious root, Rboh, ROS, poplar, genetic improvement

## Abstract

Adventitious roots (ARs) are essential for clonal vegetative propagation of woody tree species. Extensive studies have focused on phytohormone and transcription factor regulatory networks during AR development in poplar, yet the regulatory roles of reactive oxygen species (ROS) signaling during AR development remain poorly understood. In this study, we performed a genome-wide identification and systematic characterization of the *Respiratory Burst Oxidase Homolog* (*Rboh*) gene family in *Populus trichocarpa*. A total of 10 PtrRbohs were identified and phylogenetically classified into five subfamilies. Expression profiling revealed distinct tissue-specific and stress-responsive expression patterns among *PtrRbohs*. Functional validation via CRISPR/Cas9-mediated knockout showed that disruption of *PtrRbohB* significantly reduced AR length, diameter, and lateral root density in poplar. Heterologous overexpression of *PtrRbohB* in *Arabidopsis thaliana* enhanced ROS accumulation, confirming its conserved function in ROS generation. Furthermore, knockout of *PtrRohB* down-regulated the expression of several AR-positive regulators and up-regulated that of AR-negative regulators in poplar. Collectively, this study provides the direct genetic evidence that *Rboh* regulates AR formation in poplar, and identifies *PtrRbohB* as a key positive regulator of AR development. These findings enrich our understanding of woody plant AR formation and provide candidate genes for molecular breeding of poplar rooting traits.

## 1. Introduction

Adventitious roots (ARs) initiate from non-root tissues such as stems and branches, and are indispensable for clonal vegetative propagation of most woody plant species [1]. For forest trees, the capacity for AR formation directly determines the survival rate of cuttings, the popularization of elite germplasm, and the efficiency of large-scale afforestation [2]. In many woody species, poor rooting ability severely restricts cutting breeding and genetic improvement of excellent varieties [2]. Over decades, substantial progress has been achieved in exploring anatomical characteristics, physiological and biochemical changes, and hormone regulatory networks governing woody plant AR development [3]. Nevertheless, most mechanistic knowledge originates from herbaceous model plants; the molecular regulatory framework underlying woody-specific AR reprogramming remains fragmentary. In particular, how wound-triggered signal molecules coordinate cell fate transition during AR induction and initiation has not been fully elucidated, which hinders our capacity to genetically improve rooting traits in forest trees.

Multiple transcription factors and hormone-related regulatory modules controlling woody AR development have been identified in recent years. In poplar, key genes including *WOX11*, *SCR*, *LBD16*, *AIL1*, and *RR13* participate in the cell dedifferentiation, primordium initiation and outgrowth of ARs, forming a complex transcriptional regulatory cascade intertwined with auxin–cytokinin crosstalk [4,5]. Similar regulatory factors have also been characterized in other tree genera, yet these studies largely center on phytohormone and transcription factor pathways [6,7]. Increasing physiological evidence indicates that reactive oxygen species (ROS) act as vital secondary messengers upon wounding stimulus during cutting propagation, participating in cell wall remodeling and cell fate reprogramming of root founder cells [8,9,10]. Accordingly, it is reasonable to speculate that ROS-generating components may serve as critical regulatory factors for woody plant AR formation, deserving further genetic validation.

Plasma membrane-localized Respiratory Burst Oxidase Homolog (Rboh, also known as NADPH oxidase) is the primary enzyme responsible for apoplastic ROS production in plants [11]. Upon perceiving developmental or stress signals, Rboh proteins use the cytosolic nicotinamide adenine dinucleotide phosphate reduced form (NADPH) as an electron donor to catalyze superoxide anion generation, which is subsequently converted into hydrogen peroxide and other ROS molecules to modulate plant growth, morphogenesis, and stress adaptation, especially the elongation of the pollen tube and the regulation of stem cell activity [8,9]. Functional studies in herbaceous plants have demonstrated that Rboh-derived ROS modulates root-hair elongation, lateral-root emergence, cell-wall rigidity modification and hormone signal transduction [12]. For instance, *AtRbohC* is essential for root-hair tip growth in *Arabidopsis thaliana*; loss-of-function mutation leads to shortened root hairs and disturbed root apical development [13]. Recent transcriptomic studies on apple and camphor tree cuttings revealed that several *Rboh* homologs are significantly induced during AR primordia formation, implying potential links between Rboh-dependent ROS accumulation and AR development in woody plants [6,7]. However, the direct genetic functional verification of the *Rboh* gene family remains scarce in perennial woody trees.

Poplars (*Populus* spp.) represent the most important fast-growing perennial woody model tree, possessing enormous ecological value in ecological restoration and considerable economic value for timber, pulp and bio-energy industries [14]. Among poplar taxa, sections Tacamahaca (balsam poplars) are characterized by high adventitious rooting capacity, which provides superior material for cutting propagation and cross-breeding [15]. *Populus trichocarpa*, a representative species of balsam poplar, has inherently easy-to-root traits, which distinguish it from poplar species that are difficult to root. In addition, it boasts a high-quality reference genome and a mature, stable genetic transformation system [16], making *P. trichocarpa* an ideal experimental system for dissecting the genetic basis that governs the development of ARs in woody plants. In this study, we systematically identified 10 *PtrRboh* family members across the *P. trichocarpa* genome and performed comprehensive analyses of phylogeny, gene duplication, conserved structure, *cis*-elements, post-translational modification (PTM) sites and tissue/stress-responsive expression profiles. A root-preferentially expressed gene, *PtrRbohB,* was selected for further functional characterization via CRISPR/Cas9-mediated gene knockout and heterologous overexpression assays. Our results demonstrate that *PtrRbohB* positively regulates ARs growth and modulates ROS accumulation. Collectively, these findings enrich our understanding of *Rboh* functions and provide candidate genes for molecular breeding targeting poplar rooting traits.

## 2. Results

### 2.1. Identification and Phylogenetic Analysis of Rbohs

Ten Rboh family members were identified in *P. trichocarpa* based on the NADPH_Ox and NAD_binding_6 domains. According to their evolutionary relationships with *A. thaliana*, these members were named PtrRbohA through PtrRbohJ (Table S1). PtrRboh proteins varied in length from 846 aa (PtrRbohJ) to 949 aa (PtrRbohI), with molecular weights ranging from 96.7 kDa (PtrRbohJ) to 107.7 kDa (PtrRbohI) and isoelectric points between 9.10 (PtrRbohG) and 9.41 (PtrRbohF and PtrRbohH). Subcellular localization prediction indicated that all 10 PtrRbohs were membrane-localized. Among PtrRbohs, eight members possessed four transmembrane helices, PtrRbohJ/PtrRbohH had five and six transmembrane helices, respectively (Figure S1).

**Figure 1:**
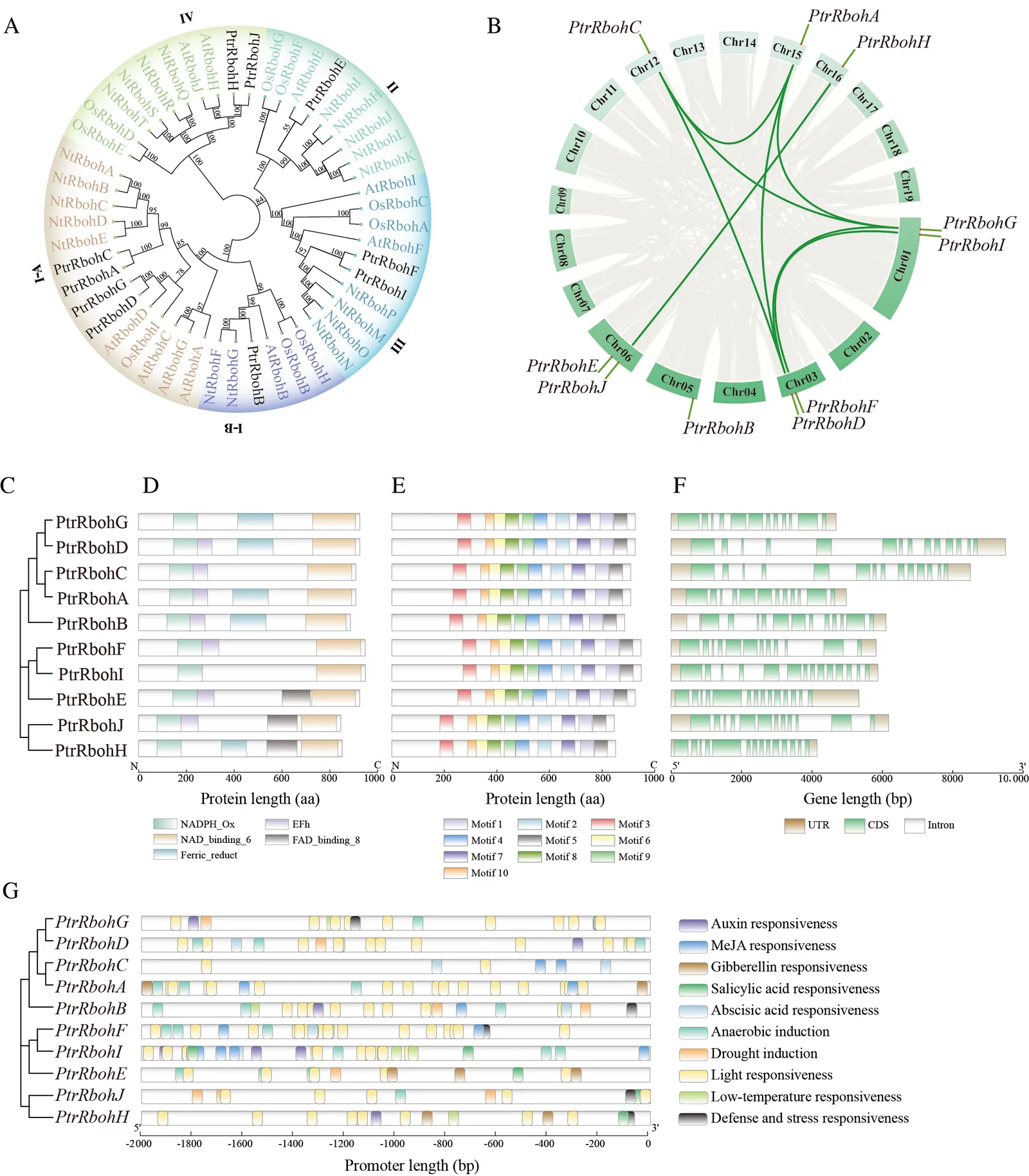
Bioinformatic characterization of PtrRbohs. (**A**) Phylogenetic relationships of Rboh proteins. These proteins are clustered into five subfamilies, each marked by a distinct color (beige, purple, green, light green, and blue). (**B**) Chromosomal distribution and synteny of PtrRbohs. Nineteen chromosomes are represented by green boxes; grey lines and green lines represent collinearity relationships of genome and PtrRbohs, respectively. (**C**) Phylogenetic analysis of PtrRbohs. (**D**) Conserved domains of PtrRbohs. (**E**) Motifs of PtrRbohs. (**F**) Gene structure of *PtrRbohs*. (**G**) Analysis of *cis*-acting elements in promoters of *PtrRbohs*.

For phylogenetic analysis, 49 Rboh protein sequences from *P. trichocarpa*, *A. thaliana*, *Oryza sativa*, and *Nicotiana tabacum* were retrieved and used to construct a phylogenetic tree (Figure 1A). These Rbohs clustered into five subfamilies: I-A comprised four PtrRbohs (PtrRbohA, PtrRbohC, PtrRbohD, and PtrRbohG); I-B comprised PtrRbohB; II comprised PtrRbohE; III comprised PtrRbohF and PtrRbohI; and IV comprised PtrRbohH and PtrRbohJ.

### 2.2. Chromosome Localization and Synteny Analysis of PtrRbohs

To investigate the duplication of *PtrRbohs*, chromosomal distribution and synteny relationships were examined. In *P. trichocarpa*, 10 *PtrRbohs* were unevenly distributed across six chromosomes (Figure 1B), namely Chr01, Chr03, Chr06, Chr12, Chr15, and Chr16. Genome-wide synteny analysis revealed eight syntenic gene pairs within the Rboh family. *PtrRbohA*, *PtrRbohC*, *PtrRbohD*, and *PtrRbohG* formed a collinear relationship comprising four genes. *PtrRbohJ* was collinear with *PtrRbohH*, and *PtrRbohI* was collinear with *PtrRbohF*. In contrast, *PtrRbohB* and *PtrRbohE* had no collinear relationship and were identified as singletons. Among these eight gene pairs, eight *PtrRbohs* were involved in eight segmental duplication events, whereas none underwent tandem duplication (Table S2), suggesting that segmental duplication serves as the primary force driving *PtrRbohs* expansion. All gene pairs had Ka/Ks values below 1 (Table S2), implying that purifying selection contributed substantially to the evolution of *PtrRbohs*.

### 2.3. Conserved Motif, Gene Structure, and Conserved Domain Analysis

To characterize the structural similarities and differences among PtrRbohs, we examined their conserved features. An evolutionary tree of the 10 PtrRbohs was constructed (Figure 1C), and conserved domains were predicted by the NCBI-CDD tool (Figure 1D). Domain analysis revealed that NADPH_Ox and NAD_binding_6 were present in all PtrRbohs, whereas the domains of the Ferric_reduct, EFh, and FAD_binding_8 varied among these proteins, and PtrRbohI simultaneously lacked these three domains. Analysis of conserved motifs showed that all PtrRbohs had 10 conserved motifs (Figure 1E and Figure S2). Analysis of gene structure showed that most PtrRbohs shared similar exon–intron organization (Figure 1F). *PtrRbohC*, *PtrRbohD*, *PtrRbohE*, *PtrRbohH*, and *PtrRbohI* had 13 introns; *PtrRbohG*, *PtrRbohA*, *PtrRbohF*, and *PtrRbohJ* had 11 introns. *PtrRbohB* had 12 introns, different from the others. The difference also appeared in the UTRs. *PtrRbohB* had three UTRs (including two separated 5′-UTRs), while the other nine *PtrRbohs* had only two UTRs. These data indicate that PtrRbohs have a close evolutionary relationship, although there are some differences in gene structure and protein characteristics.

### 2.4. Stress-, Growth-, and Development-Related Cis-Element Analysis of PtrRboh Promoters

To explore the potential functions of *PtrRbohs*, stress-, growth-, and development-related *cis*-acting elements were predicted in their promoters (Figure 1G). These elements included light-responsive, hormone-responsive (gibberellin, abscisic acid, salicylic acid, methyl jasmonate (MeJA), and auxin), and stress-responsive (drought-inducible, defense/stress-responsive, low-temperature-responsive, and anaerobic induction) elements. The positions and numbers of the 10 element types differed markedly. Light-responsive elements were present in all promoters and accounted for the largest proportion (42.23%). Most PtrRbohs had anaerobic induction- and salicylic acid-responsive elements. Auxin-responsive elements were detected only in *PtrRbohG*, *PtrRbohD*, *PtrRbohB*, *PtrRbohI*, and *PtrRbohH*. Gibberellin-responsive elements were found in *PtrRbohA*, *PtrRbohE*, and *PtrRbohH*. MeJA-responsive elements were found in *PtrRbohA*, *PtrRbohB*, *PtrRbohC*, *PtrRbohI*, and *PtrRbohF*. Drought-inducible, defense, and stress-responsive elements were detected only in *PtrRbohG*, *PtrRbohB*, *PtrRbohJ*, and *PtrRbohH,* whereas low-temperature-responsive elements occurred in *PtrRbohD*, *PtrRbohB*, *PtrRbohI*, and *PtrRbohH*. These findings suggest that *PtrRbohs* participated in multiple pathways regulating plant growth, development, and stress responses.

### 2.5. Analysis of PTM Sites in PtrRbohs

**Figure 2:**
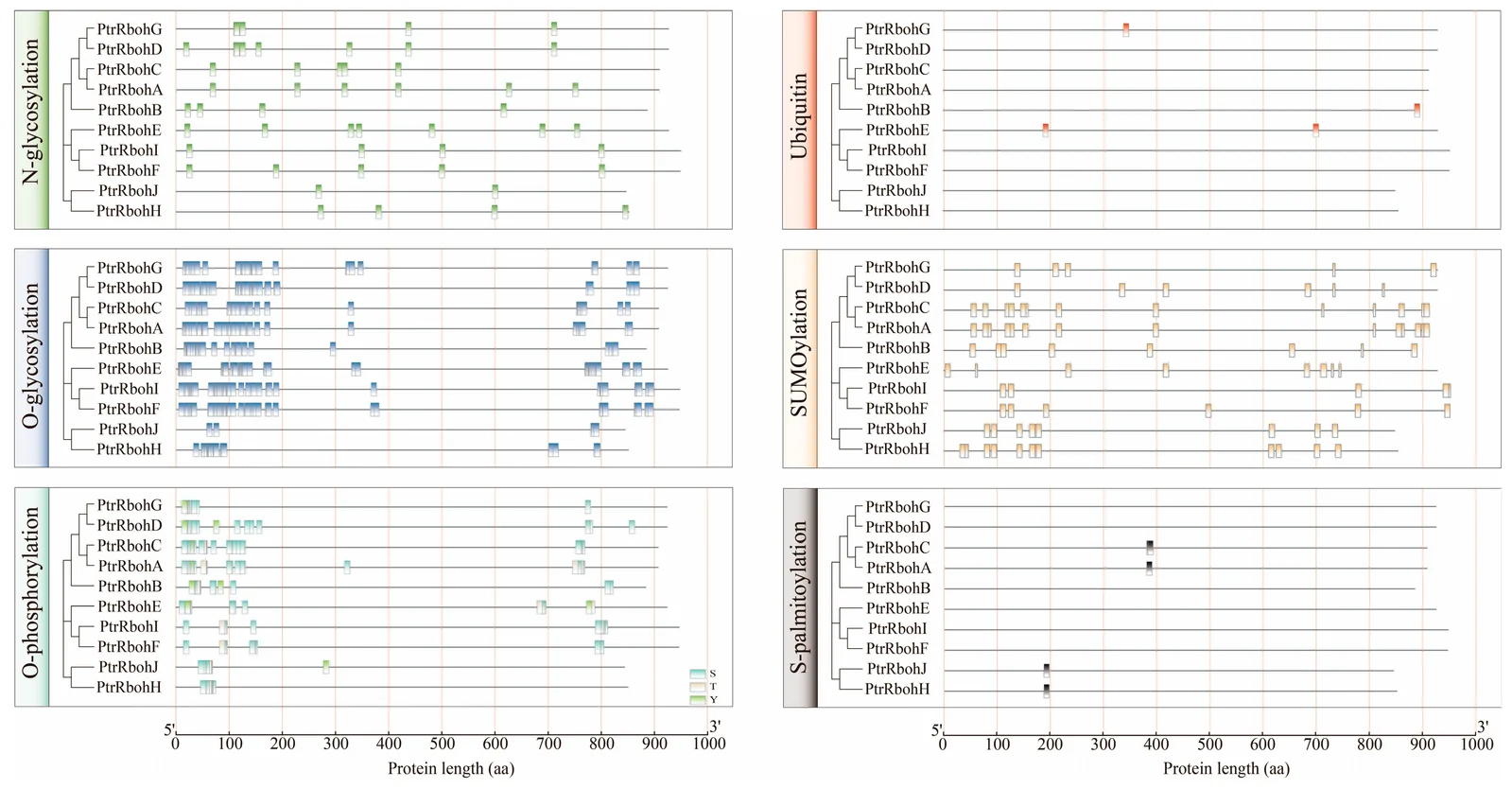
PTM site prediction of PtrRbohs. Parameter settings: N-glycosylation (threshold was set at >0.5), O-glycosylation (threshold was set at >0.5), O-phosphorylation (score was set at >0.8), ubiquitin (threshold was set at >0.5), SUMOylation (threshold was set at >0.5), and S-palmitoylation (threshold was set at >0.5).

PTMs typically serve as key regulators of Rboh activity [17]; therefore, we analyzed six PTM sites (N-glycosylation, O-glycosylation, O-phosphorylation, ubiquitylation, SUMOylation, and palmitoylation) in PtrRbohs (Figure 2, Table S3). Closely related members tend to share more similar PTM patterns, with modification sites preferentially enriched at the N- and C-termini. Among all 10 proteins, phosphorylation sites were the most abundant, with a total of 128 sites; phosphorylation site counts varied from 8 in PtrRbohJ to 22 in PtrRbohC per protein. O-glycosylation (*n* = 77, N represents the total number of modification sites, and the same applies below) and SUMOylation (*n* = 76) were the second and third most abundant PTMs, respectively, in terms of the total number of modified proteins. Ubiquitylation had the fewest sites (*n* = 4), with counts ranging from 0 (PtrRbohI, PtrRbohF, PtrRbohD, PtrRbohA, PtrRbohC, and PtrRbohH) to 2 (PtrRbohE) per protein; PtrRbohB and PtrRbohG each had one site. In addition, O-glycosylation, O-phosphorylation, and SUMOylation sites were abundantly distributed at the N- and C-termini of these proteins. The PtrRbohs had a long chain at the N-terminus and a β-sheet at the C-terminus (Figure S2), suggesting that PTM may influence protein structure and potential functions.

### 2.6. Expression Pattern Analysis of PtrRbohs

**Figure 3:**
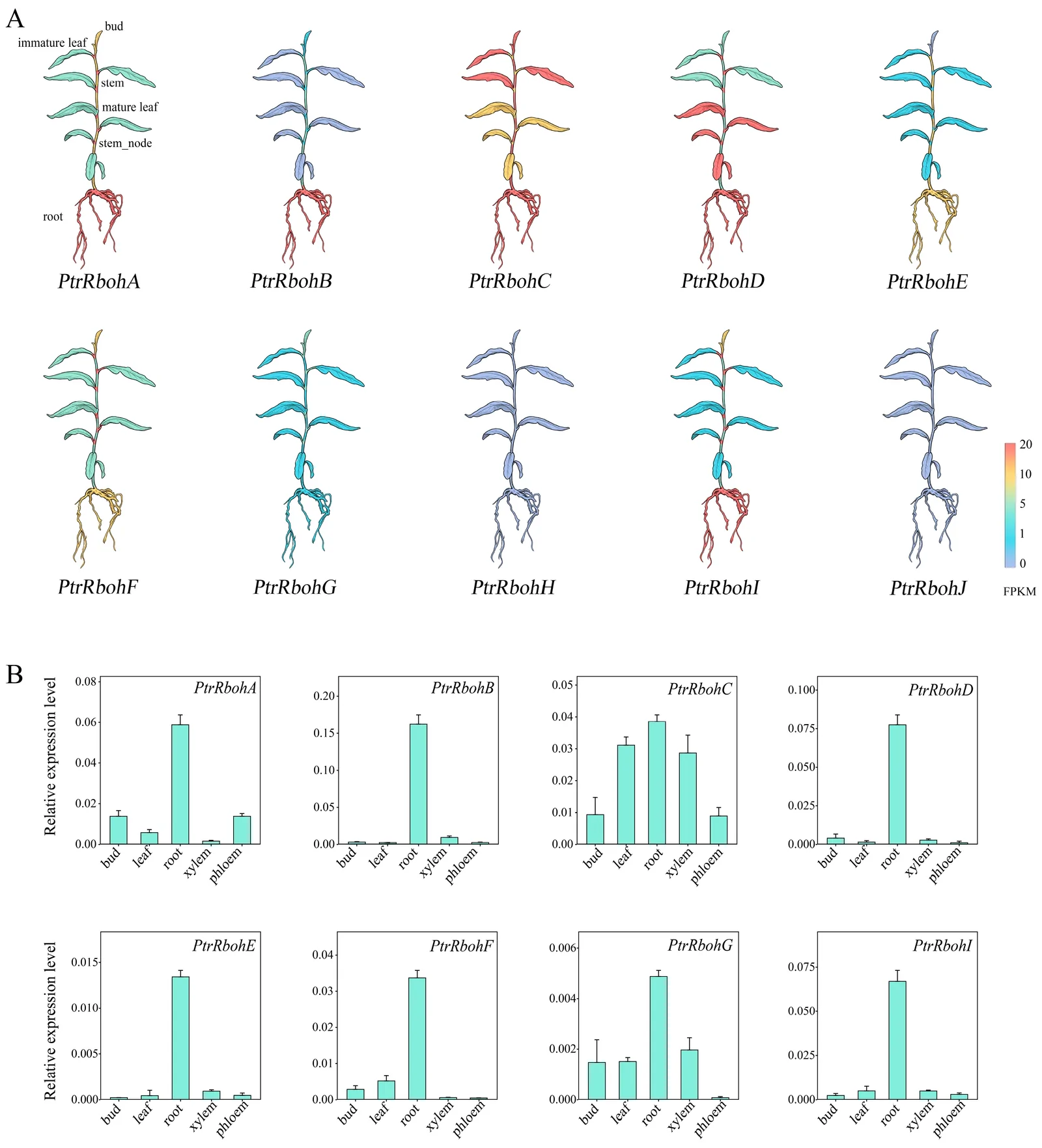
Expression pattern analysis of *PtrRbohs*. (**A**) Expression levels of *PtrRbohs* in 6 tissues: bud, immature leaf, mature leaf, stem, the node of stem, and root. (**B**) Expression levels of *PtrRbohs* in bud, leaf, root, xylem, and phloem. All experiments were performed with three biological replicates, and error bars represent the standard deviation.

To further confirm the functions of *PtrRboh* genes, expression patterns in buds, immature and mature leaves, stems, stem nodes, and roots were analyzed. The results showed significant differences (Figure 3A). *PtrRbohA* exhibited peak expression in stems and stem nodes, indicating its association with lignification. *PtrRbohB*, *PtrRbohD*, *PtrRbohE*, *PtrRbohF*, and *PtrRbohI* were preferentially expressed in roots, suggesting their involvement in root development. Additionally, RT-qPCR was conducted to analyze and test the expression patterns of *PtrRbohs* in buds, leaves, roots, xylem, and phloem (Figure 3B). The verification results are roughly similar to the transcriptome data. Six *PtrRbohs* (*PtrRbohA*, *PtrRbohB*, *PtrRbohD*, *PtrRbohE*, *PtrRbohF*, and *PtrRbohI*) showed preferential expression in roots, indicating that *PtrRbohs* may be involved in root development. Among them, *PtrRbohB* had the highest relative expression level in roots, suggesting its potential functions in root development.

### 2.7. Expression of PtrRbohs Under Drought, Salt, Heat, and Cold Stress

**Figure 4:**
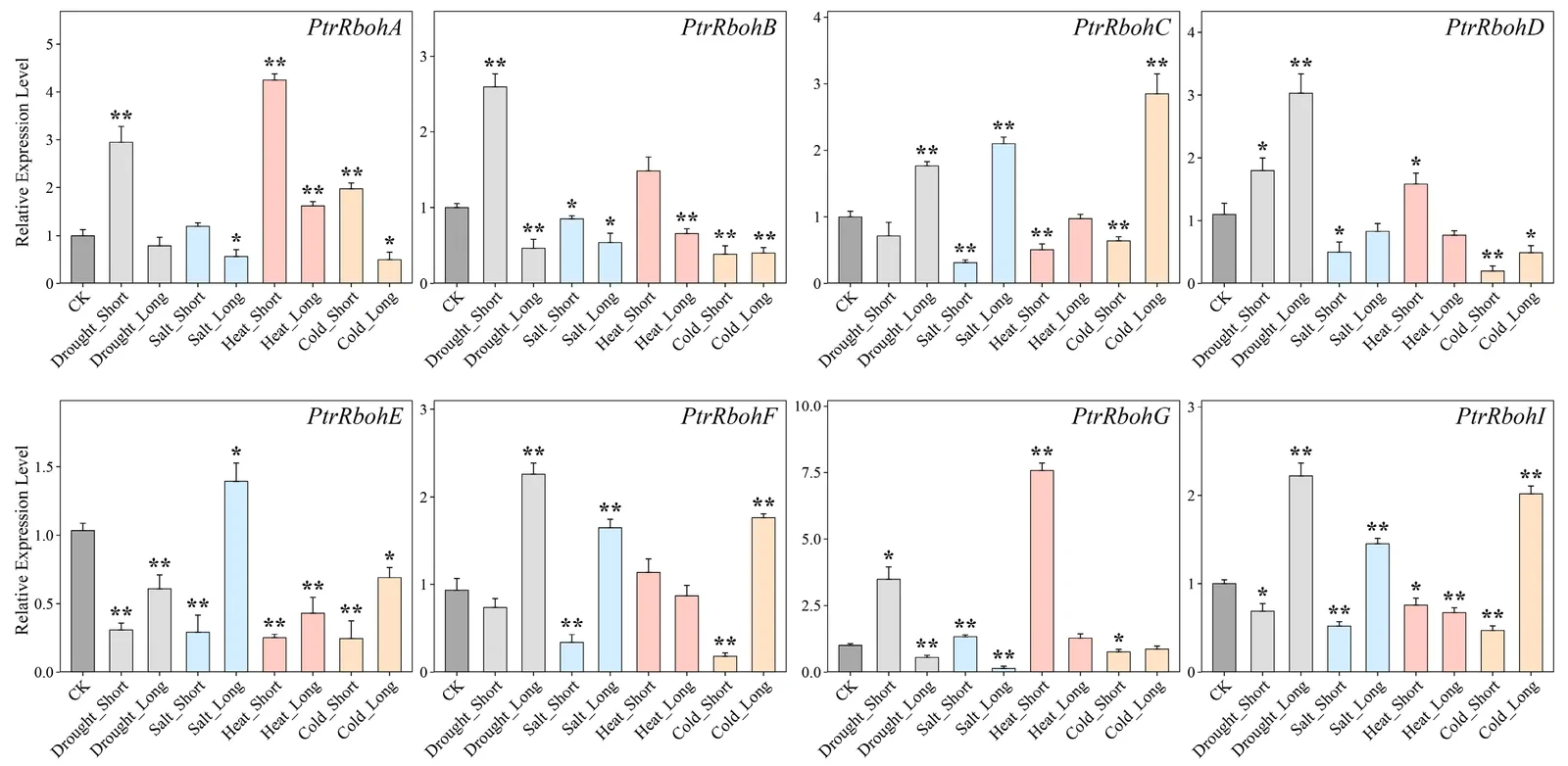
Expression levels of *PtrRbohs* in roots under drought, salt, heat, and cold stress. Eight groups of data are presented, including short- and long-term treatments for each stress treatment. All experiments were performed with three biological replicates, and error bars represent the standard deviation. Statistical significance was assessed with one-way ANOVA (* p < 0.05, ** p < 0.01).

To investigate the response of *PtrRbohs* to abiotic stresses, RT-qPCR was performed to quantify the transcript levels of all *PtrRboh* members in roots under short-term and long-term exposure to drought, salt, heat, and cold stresses (Figure 4). Under drought stress, *PtrRbohA*, *PtrRbohB*, *PtrRbohD*, and *PtrRbohG* showed significantly increased expression during short-term treatment, whereas *PtrRbohE* exhibited decreased expression under both short-term and long-term drought stress. Under 100 mM NaCl stress, the expression levels of four *PtrRbohs* (*PtrRbohC*, *PtrRbohE*, *PtrRbohF*, and *PtrRbohI*) showed time-dependent expression patterns, and *PtrRbohC*, *PtrRbohE*, *PtrRbohF*, and *PtrRbohI* showed decreased expression during short-term salt treatment but increased expression under long-term salt stress, suggesting a dynamic adaptive response to salt stress over time. For heat stress, *PtrRbohA* and *PtrRbohG* exhibited high expression levels under short-term heat stress, suggesting that these two members are involved in the early response to heat stress in poplar roots. Under long-term cold stress, *PtrRbohC*, *PtrRbohF*, and *PtrRbohI* were significantly up-regulated under long-term cold stress, indicating their potential roles in long-term cold acclimation. Collectively, *PtrRboh* family members exhibited distinct differences in expression responses to different abiotic stresses. These results also provide basic expression-level data for further exploring the roles of *PtrRbohs* in poplar stress adaptation.

### 2.8. Knockout of PtrRbohB Inhibits AR Development in P. trichocarpa

To confirm the function of *PtrRbohB*, we constructed stable transgenic Arabidopsis lines with the floral dip method, and three highly expressed lines were selected for further analysis (Figure S3A). Since PtrRbohB is annotated as an NADPH oxidase, we collected several fresh leaves from the transgenic lines for histological DAB staining analysis (Figure S3B). The results revealed that after decolorization, compared with the wild-type (WT) plants, *PtrRbohB-OE* lines displayed dark brown leaves, which indicated increased ROS accumulation in the overexpression lines. The conserved Rboh domains (Figure 1D), together with DAB staining showing ROS accumulation, collectively confirm the function of *PtrRbohB* as an Rboh.

**Figure 5:**
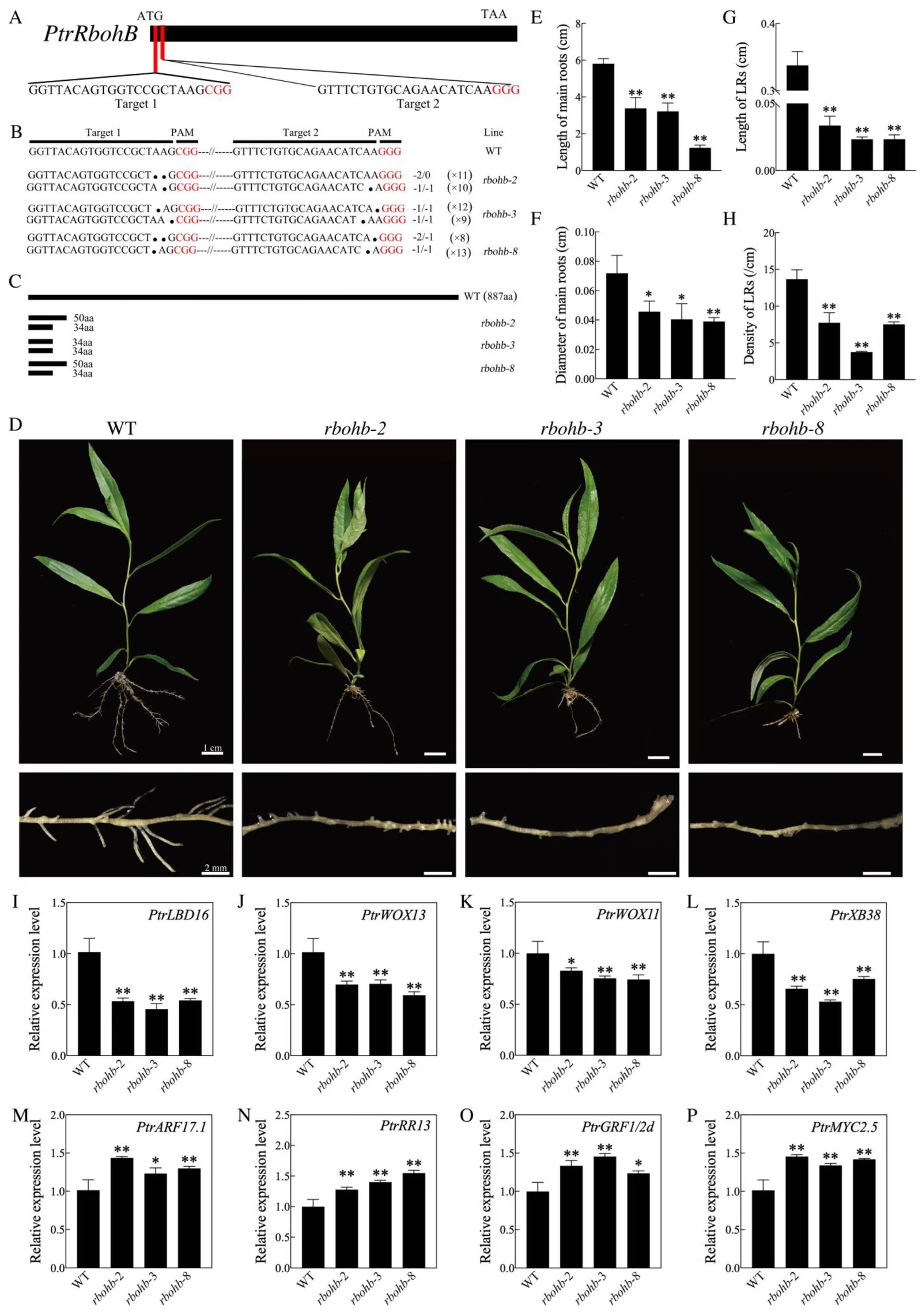
CRISPR/Cas9-induced *ptrrbohb* mutations in *P. trichocarpa*. (**A**) CRISPR/Cas9 target sites of *PtrRbohB*. Target1/2 indicate the positions of gRNA-targeted sites by different pHSE401-2gRNA vectors. (**B**) Mutation sites in *PtrRbohB*. Black text and dots represent native gRNA target sequences and deleted nucleotides, respectively. Red text denotes the PAM (protospacer adjacent motif) (**C**) Selected potential mutated PtrRbohB proteins. (**D**) Phenotypic differences of roots between WT and transgenic lines. (**E**) Length of roots. (**F**) Diameter of roots. (**G**) Length of LRs. (**H**) Density of LRs. (**I**) Expression level of *PtrLBD16.* (**J**) Expression level of *PtrWOX13*. (**K**) Expression level of *PtrXB38*. (**L**) Expression level of *PtrWOX11*. (**M**) Expression level of *PtrARF17.1*. (**N**) Expression level of *PtrRR13*. (**O**) Expression level of *PtrGRF1/2d*. (**P**) Expression level of *PtrMYC2.5*. All experiments were performed with three biological replicates, and error bars represent the standard deviation. Statistical significance was assessed with one-way ANOVA (* p < 0.05, ** p < 0.01).

To further clarify the biological function of *PtrRbohB*, we constructed several *ptrrbohb* knockout mutants. Sequencing analysis of the edited target sequences confirmed that the frameshift induced by the mutation introduced a premature stop codon, resulting in the early termination of translation (Figure 5A–C). We then selected three independent transgenic lines (*rbohb-2/3/8*) to investigate the root phenotype. Compared with WT plants, the *rbohb* mutant lines showed significantly different root system architecture. The AR length and diameter of *rbohb* lines decreased significantly, as did the length and density of lateral roots (Figure 5D–H). These results indicate that knockout of *PtrRbohB* significantly inhibits AR development. To further explore the role of *PtrRbohB* in AR development, RT-qPCR was carried out to examine analyze the expression of several marker genes associated with AR development. The expression levels of positively regulated genes (*PtrLBD16*, *PtrWOX13*, *PtrWOX11*, and *PtrXB38*) decreased significantly relative to WT (Figure 5I–L). In contrast, the expression levels of negatively regulated genes (*PtrARF17.1*, *PtrRR13A*, *PtrGFR1/2d*, and *PtrMYC2.5*) were significantly higher than those in WT (Figure 5M–P).

## 3. Discussion

### 3.1. Identification and Evolutionary Conservation and Divergence of the PtrRbohs

In this study, 10 *PtrRboh* family members were identified in the *P. trichocarpa* genome and phylogenetically divided into five subfamilies (I-A, I-B, II, III, IV). The number of *Rboh* family members varies widely across plant lineages: 10 in *A. thaliana* [18], nine in rice [19], 20 in tobacco [20], and up to 40 in bread wheat [21]. Notably, *P. trichocarpa* harbors the same number of *Rboh* genes as *A. thaliana*, despite over 100 million years of independent evolution between woody angiosperms and herbaceous *Brassicaceae* [22]. Synteny analysis revealed that segmental duplication, rather than tandem duplication, was the dominant driving force for PtrRboh family expansion (Figure 1B). All collinear gene pairs had Ka/Ks values far below 1, indicating that purifying selection has acted to maintain the core enzymatic functions of Rboh proteins during poplar evolution. Meanwhile, all PtrRbohs contain the two core catalytic domains, NADPH_Ox and NAD_binding_6, as well as 10 conserved motifs (Figure 1D), reflecting high evolutionary conservation of basic Rboh function. Nevertheless, noticeable structural divergence was observed among subfamilies: intron number ranged from 11 to 13, the number of transmembrane helices also varied from 4 to 6, and the distribution of predicted sites of PTMs (Figure 1F, Figure 2 and Figure S2). These structural divergences, especially in promoter regions and PTM sites, provide the molecular basis for the differential expression patterns and functional specialization of *PtrRboh* family members.

### 3.2. Functional Divergence of PtrRbohs in Growth, Development and Stress Responses

Gene expression patterns are tightly linked to their biological function. Tissue expression profiling showed that *PtrRboh* members exhibited distinct tissue specificities (Figure 3), implying extensive functional differentiation. *PtrRbohC* was preferentially expressed in buds and leaves, suggesting involvement in leaf development and morphogenesis. *PtrRbohA* and *PtrRbohI* showed peak expression in stems, consistent with potential roles in xylem lignification and secondary cell wall formation. Additionally, *PtrRbohB*, *PtrRbohD*, *PtrRbohE*, *PtrRbohF*, *and PtrRbohI* were abundantly expressed in roots, indicating their participation in root system development and function. For abiotic stress responses, these *PtrRbohs* also showed differentiated response patterns under drought, salt, heat and cold stresses (Figure 4). Most *PtrRbohs* were slightly up-regulated under salt stress, suggesting a conserved role for RBOH-mediated ROS signaling in salt stress. In contrast, *PtrRbohA* and *PtrRbohB* were induced by short-term drought, while *PtrRbohC*, *PtrRbohF* and *PtrRbohI* were up-regulated under long-term cold stress. This functional divergence is consistent with the diverse distribution of *cis*-acting elements in promoter regions (Figure 1G). Notably, all stress expression assays in this study were performed on wild-type plants; the stress response of *PtrRbohB* knockout lines will be investigated in our follow-up work. Taken together, these *PtrRbohs* have undergone functional divergence during evolution, with different members participating in distinct biological processes of growth, development and stress adaptation.

### 3.3. PtrRbohB Is a Key Positive Regulator of AR Formation

Among the root-abundant *PtrRbohs*, *PtrRbohB* showed the most prominent root-specific expression pattern, and its promoter harbors auxin-responsive elements closely associated with AR induction, indicating that *PtrRbohB* may be a key regulator of poplar AR development. Genetic evidence from CRISPR/Cas9-mediated knockout further confirmed this function; knockout of *PtrRbohB* significantly reduced AR length and diameter, as well as lateral root length and density, demonstrating that *PtrRbohB* positively regulates multiple developmental stages of ARs, including induction, initiation and elongation.

Previous transcriptomic studies in apple and poplar cuttings have shown that several *Rboh* homologs are significantly up-regulated during AR primordium formation, and exogenous H_2_O_2_ treatment can promote AR formation [23,24]. In *Arabidopsis*, RBOH-derived ROS has been proven to facilitate lateral root emergence by modifying cell wall properties [25]. Previous studies identified 10 Rboh members in *Populus yunnanensis* and analyzed their potential reactive oxygen production functions and protein interaction networks [26]; however, the biological function of *Rbohs* remained unclear, and direct genetic evidence for *Rboh* involvement in AR development in woody plants has been lacking. This study provides direct genetic evidence that Rboh protein regulates AR development in poplar, a model woody species. From a forest tree biology perspective, AR formation depends on reprogramming of differentiated somatic cells to acquire root meristem identity, and cell wall remodeling is a key step in this process [2]. The function of *PtrRbohB* in AR regulation suggests that RBOH-mediated ROS signaling may be involved in cell wall remodeling and somatic cell fate transition during poplar AR formation [2].

### 3.4. Potential Regulatory Pathway of PtrRbohB During AR Formation

RBOH proteins are the primary enzymes responsible for apoplastic ROS production in plants [8,27]. In this study, heterologous overexpression of *PtrRbohB* in *Arabidopsis* resulted in increased H_2_O_2_ accumulation in leaves as shown by DAB staining (Figure S3B), confirming that PtrRbohB retains the conserved function of catalyzing ROS production. In fact, ROS can regulate the activity of cell wall-modifying methylesterases, expansins and glycoside hydrolases, adjusting cell wall rigidity and extensibility, thereby promoting expansion of root founder cells and breakthrough of root primordia through surrounding tissues [28]. This is consistent with the finding that cell wall remodeling-related genes are highly expressed in the early stage of poplar AR formation [5]. Moreover, ROS as signaling molecules may regulate the expression of key AR-related transcription factors. RT-qPCR results showed that knockout of *PtrRbohB* significantly down-regulated the expression of AR positive regulators *PtrLBD16*, *PtrWOX11*, *PtrWOX13* and *PtrXB38*, and up-regulated negative regulators such as *PtrARF17.1* and *PtrRR13A* (Figure 5I–N). We speculate that PtrRbohB-derived ROS may modulate the expression or activity of these transcription factors, either directly or through crosstalk with auxin signaling pathways, thereby regulating AR formation. However, the upstream regulatory mechanisms of *PtrRbohB* and the direct downstream targets of ROS remain to be further experimentally verified. In addition, the PTM sites and *cis*-acting elements in this study are based on bioinformatics predictions, and their actual functions need to be confirmed by subsequent experiments. Collectively, this study identifies *PtrRbohB* as a key regulator of poplar AR development and lays a foundation for deciphering the ROS regulatory network that controls adventitious rooting in woody plants.

## 4. Materials and Methods

### 4.1. Identification and Phylogenetic Analysis of Rbohs in P. trichocarpa

To identify Rbohs in *P. trichocarpa*, 10 Rboh protein sequences from *A. thaliana* were used to blast in Phytozome (https://phytozome-next.jgi.doe.gov/, accessed on 1 July 2025). After removing redundant and incomplete sequences, 10 candidates were obtained and analyzed by NCBI-CDD (https://www.ncbi.nlm.nih.gov/Structure/cdd/wrpsb.cgi, accessed on 1 July 2025). Sequences containing the NADPH_Ox (PF08414) and NAD_binding_6 (PF08030) domains were identified as candidate Rbohs [29].

Protein sequences of Rbohs in three species (*A. thaliana*, *O. sativa*, and *N. tabacum*) were retrieved from NCBI (https://ncbi.nlm.nih.gov/, accessed on 3 July 2025) and used for phylogenetic analysis. The tree was visualized and annotated with MEGA 5.2 and iTOL (https://itol.embl.de, accessed on 7 July 2025) according to the previous research [30].

### 4.2. Chromosomal Localization and Synteny Analysis of PtrRbohs

The localization information for *PtrRbohs* was extracted from the genome file. Duplicated gene pairs were analyzed using MCScanX in TBtools (v2.441), and values of Ka/Ks were calculated with a simple Ka/Ks calculator in TBtools (v2.441) . The location and duplication relationship were visualized according to the previous research [31].

### 4.3. Conserved Domain, Conserved Motif, and Gene Structure Analyses of PtrRbohs

Conserved motifs of PtrRbohs were predicted by MEME (https://meme-suite.org/meme/, accessed on 27 July 2025) tool [32]. Conserved domains were identified by NCBI-CDD, and the gene structure information was analyzed based on the genome files [33]. Motifs, domain annotation, and gene structure information were visualized using the basic BioSequence Viewer in TBtools (v2.441) function according to the previous research [34].

### 4.4. Promoters Cis-Acting Elements and PTM Site Prediction Analysis of the PtrRboh

The 2 kb promoters were extracted from the *P. trichocarpa* genome and used for *cis*-elements prediction via PlantCARE (https://bioinformatics.psb.ugent.be/webtools/plantcare/html/, accessed on 27 August 2026). The identified *cis*-acting elements were visualized by the basic biosequence viewer [35]. For the PTM site prediction analysis, the detailed prediction method can be found in our previous research [36].

### 4.5. Expression Analysis of PtrRbohs

To determine the expression patterns of *PtrRbohs*, transcriptome data of six tissues (bud, immature leaf, mature leaf, stem, stem node, and roots) were downloaded from Phytozome (https://phytozome-next.jgi.doe.gov/, accessed on 1 September 2026) and visualized with TBtools (v2.441) [37]. Quantitative real-time PCR (RT-qPCR) was performed for five tissues (bud, leaf, root, xylem, and phloem) from 4-month-old poplar seedlings to verify the transcriptome data.

### 4.6. Plant Materials and Acquisition of Transgenic Lines

Tissue-cultured seedlings of *P. trichocarpa*, preserved in the laboratory. To obtain *ptrrbohb* mutant poplars, the knockout CRISPR/Cas9 vector was constructed as our previous description [38]. Stem segments of sterile tissue culture seedlings were used as transformation explants. To obtain overexpressed *A. thaliana*, the CDS of *PtrRbohB* was cloned into the pCAMBIA1300 vector for genetic transformation (primers are listed in Table S4). Seeds were screened by hygromycin selection, and quantitative screening was performed by the previous protocol [38]. *Populus trichocarpa* (Nisqually-1) seedlings, preserved in the laboratory, were cultured in conditions of 25 ± 2 °C, 70 ± 10% relative humidity, 5000 lux light intensity, and a 16 h light/8 h dark cycle.

### 4.7. Stress Treatment and RT-qPCR

Three-month-old seedlings of *P. trichocarpa* were used for stress treatment as described previously [39]. Both short- and long-term treatments were applied to each stress condition. Under drought treatment, plants were grown without additional watering for 7 days and 14 days. Under salt stress, plants were treated with 100 mM NaCl solution watering in the tray. Roots were collected after 24 h and 7 days. Under heat stress, plants were treated at 39 °C for 12 h and 7 days. Under cold stress, plants were subjected to 4 °C (night) and 12 °C (day) for 24 h or 7 days. Total roots were collected for RNA extraction and RT-qPCR according to the previous study [40]. *PtrActin2* (Potri.001G309500) served as the internal reference gene (Table S4). All experimental procedures, including RNA integrity validation, DNase digestion, reverse transcription, qPCR reaction setup, primer amplification efficiency calibration, target amplicon length control and thermal cycling program, were strictly performed following the corresponding manufacturer protocols.

### 4.8. Phenotype Measurement and DAB Staining

Twenty-day-old transgenic and WT poplars were used for root phenotype measurement according to our previous study [36]. Leaves of transgenic *A. thaliana* were homogenized in DAB staining solution, which contained 1 mg·mL^−1^ diaminobenzidine, PBS solution, and 95% alcohol. The vacuum treatment of the blades was performed for five minutes. After 4–5 h of shaking at 80–100 rpm, leaves were removed in 95% alcohol for decolorization, and the images were recorded [41].

### 4.9. Statistical Analysis

All experiments were repeated at least three times. Statistical analysis was performed using SPSS (19.0). Differences among groups were assessed by one-way ANOVA followed by Duncan’s multiple range test. Significance levels are indicated as * *p* < 0.05 and ** *p* < 0.01.

## 5. Conclusions

In this study, 10 *Rboh* gene family members were systematically identified in the *P. trichocarpa* genome and phylogenetically divided into five subfamilies. Evolutionary analysis revealed that segmental duplication and purifying selection were the main drivers of *PtrRboh* family expansion and functional conservation. Structural analyses showed that all PtrRboh proteins harbored conserved core catalytic domains, while divergent gene structures, *cis*-regulatory elements and post-translational modification sites provided the molecular basis for functional differentiation among family members. Expression profiling confirmed that *PtrRboh* members participate in diverse developmental processes and abiotic stress responses, with five members showing root-preferential expression patterns. Notably, the most root-specific member, *PtrRbohB*, was functionally validated as a positive regulator of AR formation in poplar. Knockout of *PtrRbohB* significantly inhibited AR development and altered the expression of key AR-related transcription factors. Overall, these findings provide valuable insights into the biological functions of woody plant *Rboh* genes and lay a foundation for molecular breeding targeting rooting efficiency in poplar and related forest trees.

## Data Availability

The original contributions presented in this study are included in the article/Supplementary Material. Further inquiries can be directed to the corresponding authors.

## Supplementary Materials

The following supporting information can be downloaded at: https://www.mdpi.com/article/10.3390/ijms27208982/s1.
