# Supplementary material for: Genome-Wide Identification and Characterization of the Rboh Gene Family in Populus trichocarpa Reveals PtrRbohB Positively Regulates Adventitious Root Development
Source: Int J Mol Sci. 2026 Oct 10;27(20):8982. doi: 10.3390/ijms27208982 (PMC13704538; doi:10.3390/ijms27208982)
Supplement: Supplementary file 1 [file ijms-27-08982-s001.zip › Supplementary Figures-260919.pdf]

Supplementary Figures

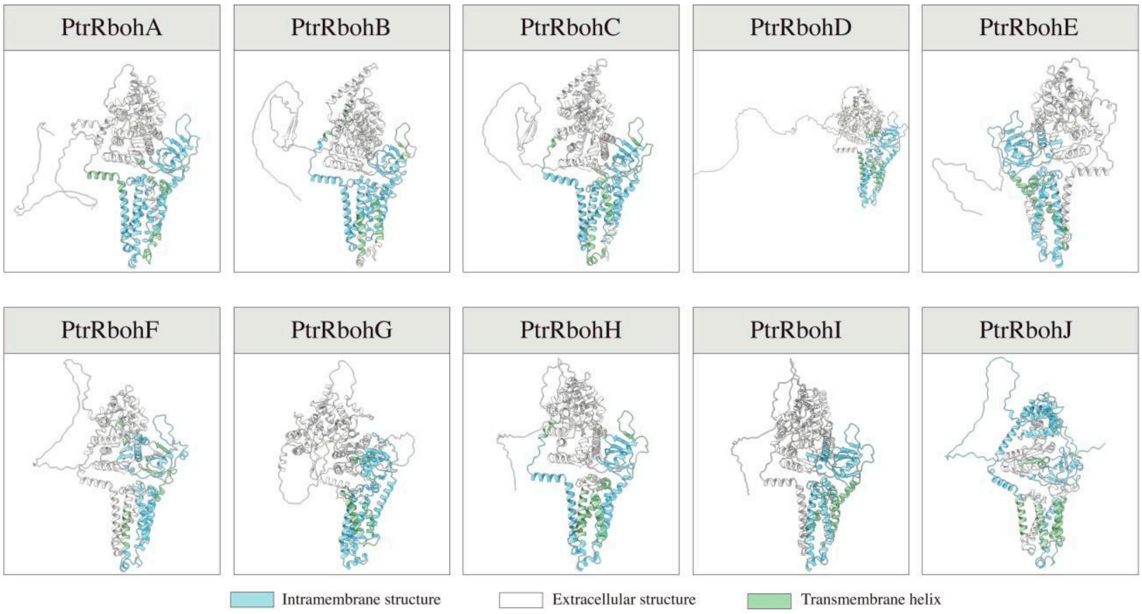

Figure S1. Tertiary structure model of PtrRbohs.

The blue part represents the intramembrane structure, the white part represents the extracellular structure, and the green part represents the transmembrane helix.

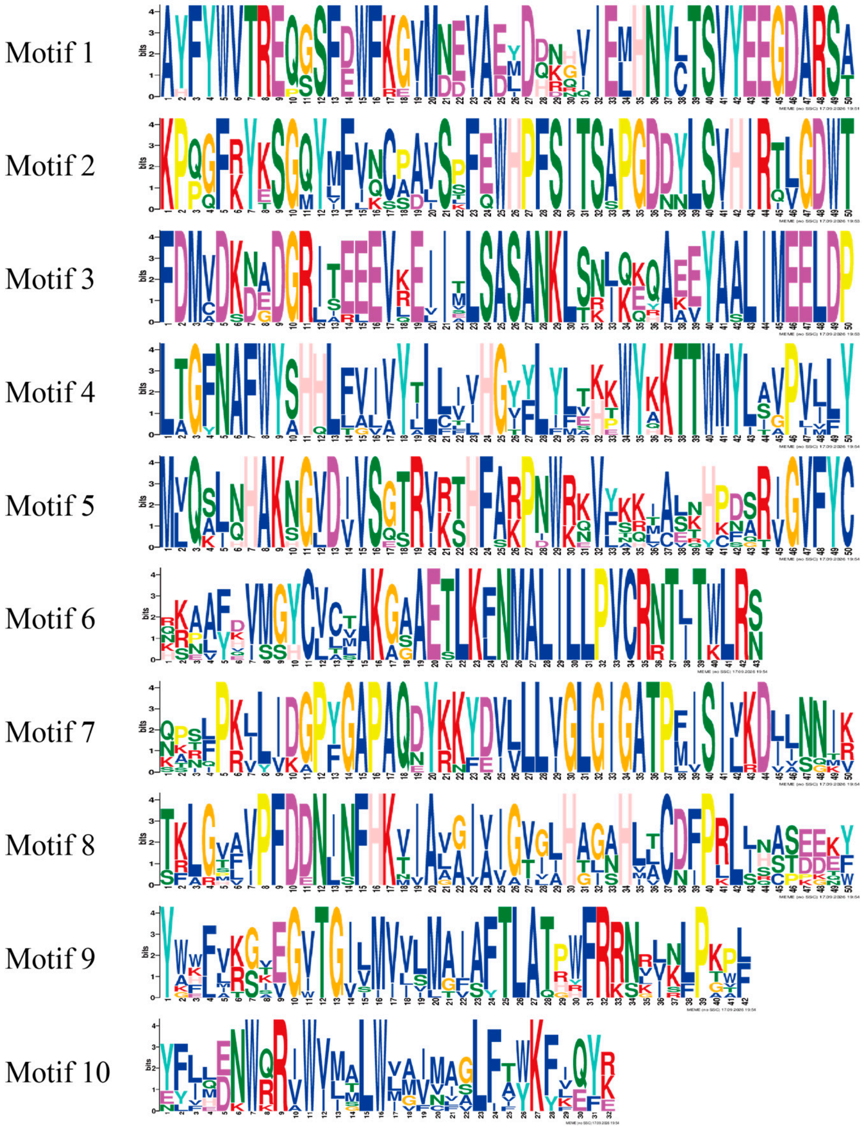

Figure S2. Conserved motifs of PtrRbohs.

The colored letters represent amino acid sites, and the numbers below them are the motif length. Bit values on the left represent the conservatism level of motifs.

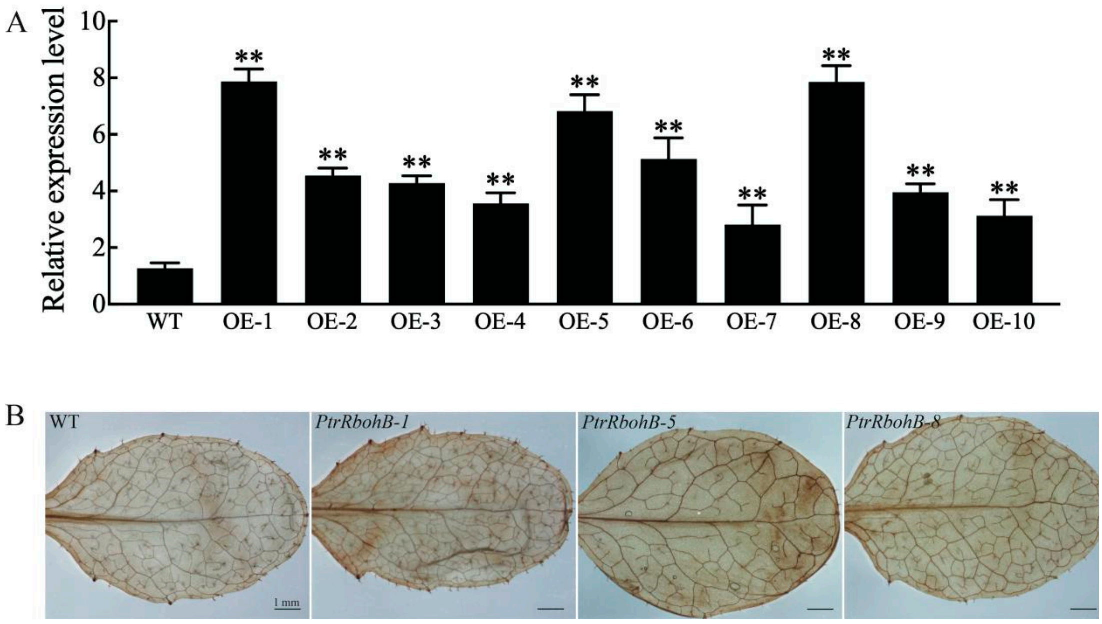

Figure S3. Phenotypic analysis of *PtrRbohB* overexpression in *A. thaliana*.

(A) Expression level analysis of *PtrRbohB* in T3 transgenic *A. thaliana*. All experiments were conducted with three biological replicates, and the error bars indicate the standard deviation. The values were statistically analyzed by one-way ANOVA, and ** represents extremely significant differences ($p < 0.01$). (B) DAB staining of 3-week-old leaves from *PtrRbohB-OE* transgenic plants. Bar=1 mm.
